# Letter to the editor regarding: ‘Construction of a predictive model for relapse of primary autoimmune hemolytic anemia: a retrospective cohort study’

**DOI:** 10.1080/07853890.2025.2523566

**Published:** 2025-06-23

**Authors:** Rong Zhou, Mengyuan Shen, Jiannong Wu

**Affiliations:** a The First School of Clinical Medicine, Zhejiang Chinese Medical University, Hongzhou, Zhejiang, China; b The First Affiliated Hospital of Zhejiang Chinese Medical University (Zhejiang Provincial Hospital of Traditional Chinese Medicine), Hongzhou, Zhejiang, China

To the editor

We read with great interest the article by Pan Li et al. titled ‘Construction of a predictive model for relapse of primary autoimmune hemolytic anemia: a retrospective cohort study’ [1]. The nomogram model constructed in this study can effectively predict the risk of relapse within one year for patients with primary AIHA, which helps in the early identification of high-risk patients and optimization of treatment strategies. However, we have some clinical considerations about this article.

Firstly, the necessity of subgroup analysis. There are three subtypes of primary AIHA, and the pathological mechanisms and treatment plans of different subtypes vary significantly [2], Additionally, the treatment plan usually needs to be adjusted accordingly based on individual factors. This study only focus on overall analysis of all patients with AIHA, which may not be sufficient to comprehensively assess the risk of relapse within one year after remission of primary AIHA. Future research could conduct subgroup analyses based on classification, whether first-line hormone treatment was used, and characteristics of drug resistance, to enhance the applicability and comprehensiveness of the study.

Secondly, there are still shortcomings in the selection of variables included in this study. This study analyzed 22 risk factors for relapse, and the variables included largely relied on single test results and qualitative results of events (such as hemoglobin at admission, whether glucocorticoids were used, and DAT positive results), making it difficult to fully present their dynamic characteristics and impact on the predictive model results. It is also important to consider including more dynamic indicators, such as the duration of first-line glucocorticoid treatment and the rate of change of Hb after therapy [3]. Additionally, monitoring the frequency of follow-up visits could provide a preliminary assessment of patient compliance. Furthermore, as the authors mentioned, DAT results (C3 and IgG) may change over time, and the frequency of C3 and IgG and their trends may more accurately reflect the risk of relapse for patients.

Furthermore, sample issue is a significant limitation of this study. The data came from only two hospitals located in the same city, with a lack of severe or fatal cases. The differences in region-related medical resources may affect the outcome and Severe AIHA patients more emphasis on personalized treatment strategies [4], Moreover, this study lacks the support of an external independent database. It is suggested to combine multi - center online data from different geographical locations to break geographical restrictions and expand sample data.

Finally, this study is based on retrospective data over a one-year period. Considering the close relationship between disease relapse and time factors, future research designs should adopt prospective research methods and extend the follow-up period to obtain more comprehensive data. This will help improve the accuracy of the relapse model assessment and broaden clinical applicability.

In summary, the author team has provided a machine learning-driven quantitative assessment model for predicting the relapse risk of primary autoimmune hemolytic anemia. We hope that the above points will be recognized and adopted to further enhance the clinical applicability of the research results.

## Data Availability

Data sharing is not applicable to this article as no new data were created or analyzed in this study.
